# Supplementary material for: Cholinergic signals preserve haematopoietic stem cell quiescence during regenerative haematopoiesis
Source: Nat Commun. 2022 Jan 27;13:543. doi: 10.1038/s41467-022-28175-1 (PMC8795384; doi:10.1038/s41467-022-28175-1)
Supplement: Supplementary file 1 — Supplementary Information [file 41467_2022_28175_MOESM1_ESM.pdf]

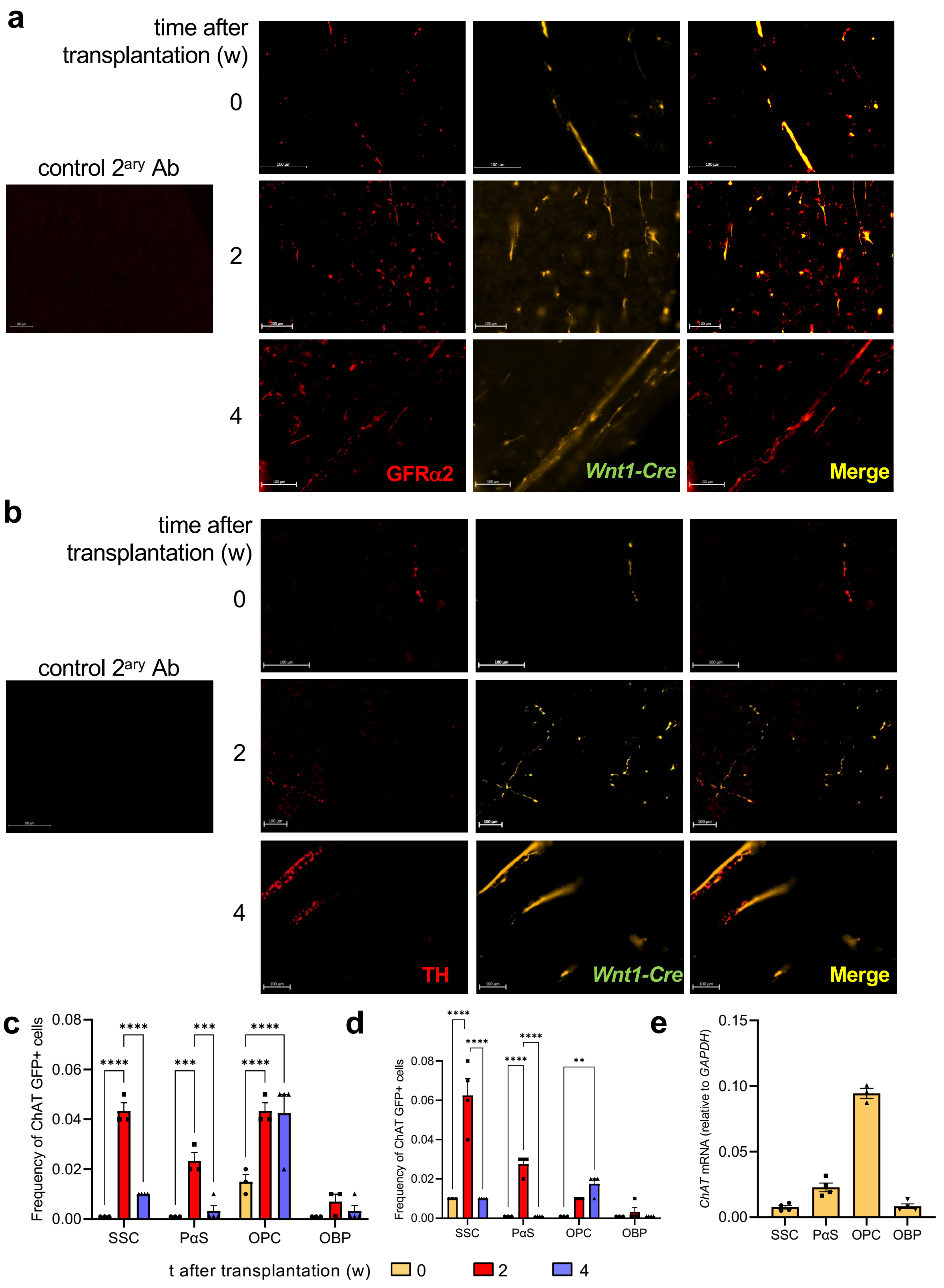

Supplementary Figure 1, related to Figure 1. Increased cholinergic and reduced noradrenergic BM innervation after transplantation. (**a**, **b**) Immunofluorescence (red) of (**a**) cholinergic (GFRα2$^+$; *N*=4,5,9) or (**b**) noradrenergic (tyrosine hydroxylase, TH$^+$; *N*=4,8,6) nerve fibres genetically marked (green) in *Wnt1Cre2;Ai14D* mice before or 2-4w after transplantation. **(c-d)** Frequency of *ChAT*- GFP$^+$ cells among BM PDGFRα+Sca1- skeletal stem cells (SSC), PDGFRα+Sca1+ (PαS) cells, PDGFRα-CD51+Sca1+ bone-lining osteoprogenitors (OPCs) or PDGFRα-CD51+Sca1- osteoblast precursors (OBPs) genetically traced in the endosteal **(c)** and central **(d)** BM of *ChAT-Gfp* mice (*N*=3-4). **(e)** mRNA expression of *ChAT* in sorted osteolineage cells of WT mice (*N*=4). (**c**-**e**) Data are mean of biological replicates ± SEM. **p<0.01, ***p<0.001. ANOVA and Tukey's multiple comparisons test.

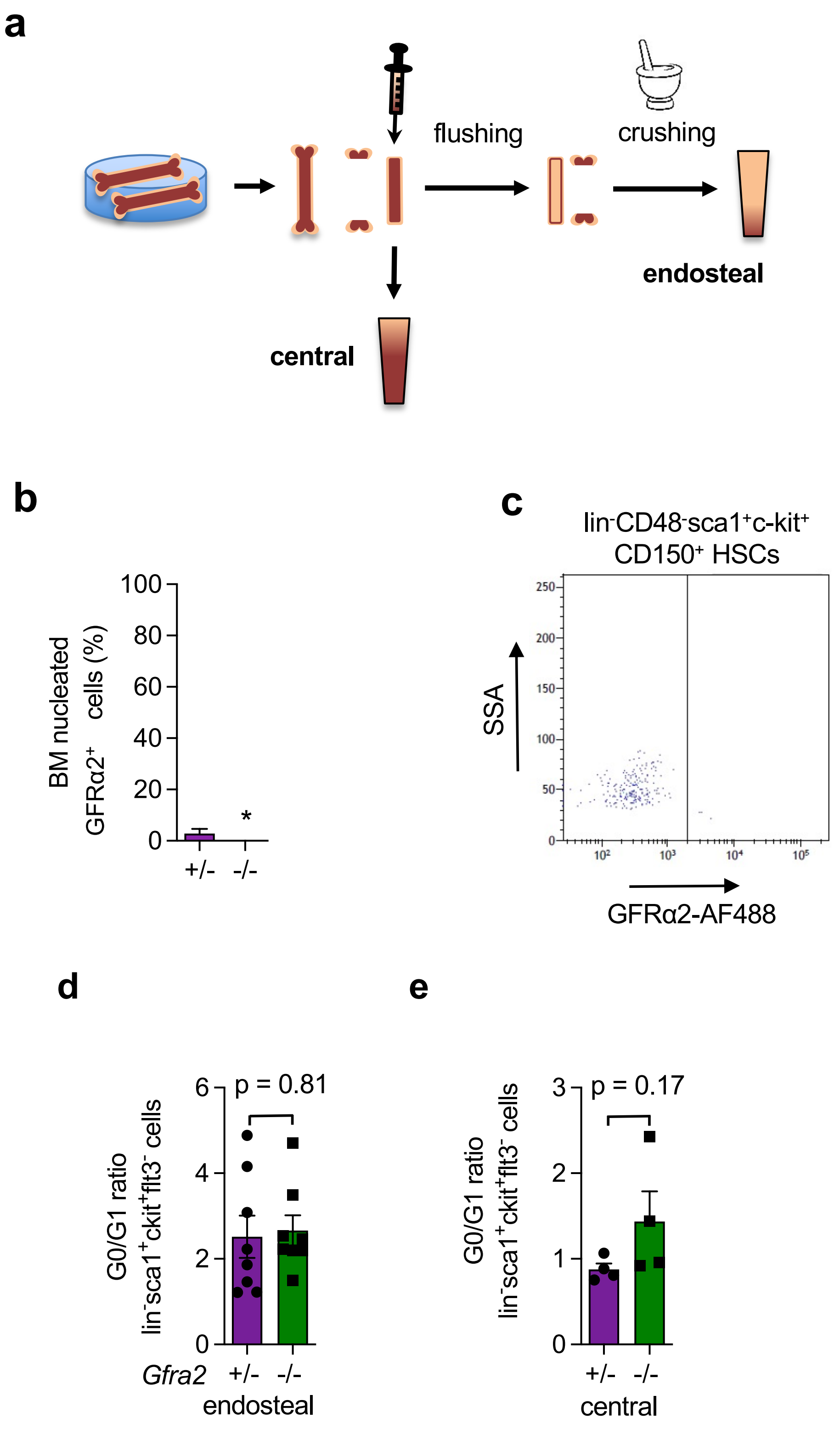

Supplementary Figure 2, related to Figure 2. Non-cell-autonomous HSC regulation by GFRα2. (**a**) Scheme showing the protocol to enrich for endosteal and central BM fractions. (**b**) Frequency of BM nucleated cells labelled with anti-GFRα2 antibody by flow cytometry in *Gfra2*$^{+/-}$ mice and *Gfra2*$^{-/-}$ mice (*N*=4; *P*=0.02). (**c**) Representative flow cytometry plot showing lack of GFRα2 in lin^{-}sca1^{+}ckit^{+} (LSK) CD48^{-} CD150^{+} HSCs. (**d-e**) Transplantation into WT mice reverts the decreased endosteal HSC quiescence observed in donor *Gfra2*$^{-/-}$ mice. Ratio of the frequencies of LSK flt3^{-} HSCs in the G0 or G1 phases of the cell cycle (LSK) isolated from the endosteal (**d**, *N*=8) or central (**e**, *N*=4) BM of WT mice 16w after lethal irradiation and transplantation into WT mice. Data are mean of biological replicates ± SEM. *P < 0.05. Unpaired two-tailed *t* test.

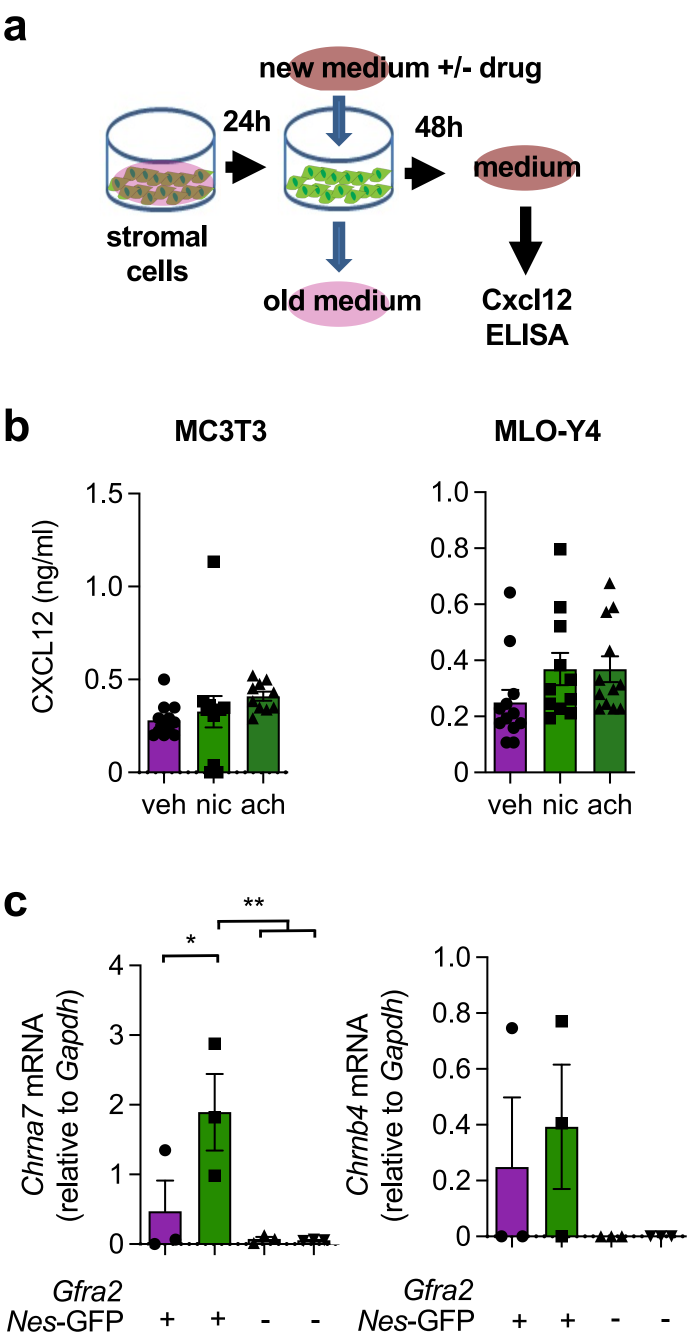

Supplementary Figure 3, related to Figure 3. Cholinergic signalling regulates HSC function through the microenvironment. (**a**) Experimental design used for cell culture and *Cxcl12* ELISA. (**b**) CXCL12 secretion by MC3T3-E1 (*N*=12,12,10) and MLO-Y4 (*N*=12,11,12) murine BM stromal cells treated with nicotine (nic), acetylcholine (ach) (10 μM) or vehicle (veh) for 24h. (**c**) Expression of nicotinic receptors α_7 (*Chrna7*) and β_4 (*Chrnb4)* (Q-PCR) in sorted BM stromal ($CD45^-CD31^-Ter119^-$) *Nes*-$GFP^{+/-}$ cells (*N*=3). (**b,c**) Data are mean of biological replicates ± SEM. One-way ANOVA and Bonferroni comparisons. *$P < 0.05$; **$P < 0.01$.

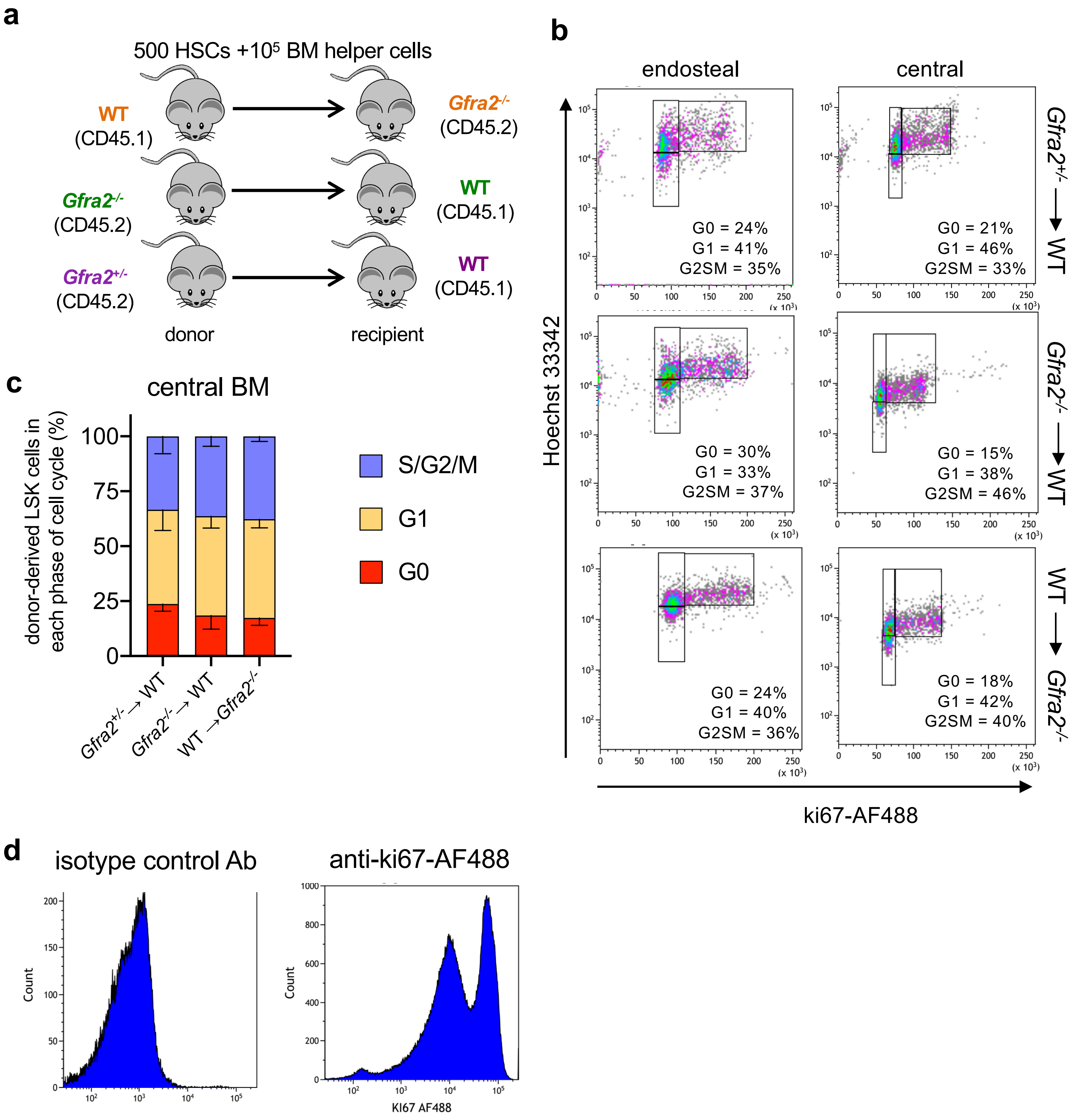

Supplementary Figure 4, related to Figures 3 and 4. Cholinergic-deficient niche renders endosteal HSCs more proliferative. (**a**) Scheme illustrating experimental design of long-term transplantation of 500 endosteal lin⁻sca1⁺ckit⁺ (LSK) CD150⁺CD48⁻ HSCs along with 10^5 BM helper cells into lethally-irradiated mice of indicated genotype. (**b**,**c**) Representative flow cytometry diagrams (**b**) and cell cycle analysis of central BM LSK cells 4 weeks after transplantation (*Gfra2*$^{+/-}$ → WT, *N*=3; *Gfra2*$^{-/-}$→ WT, *N*=3; WT →*Gfra2*$^{-/-}$, *N*=4). ANOVA and Tukey's multiple comparisons test. **(d)** Representative histograms displaying isotype control antibody (Ab) and ki67 stainings.

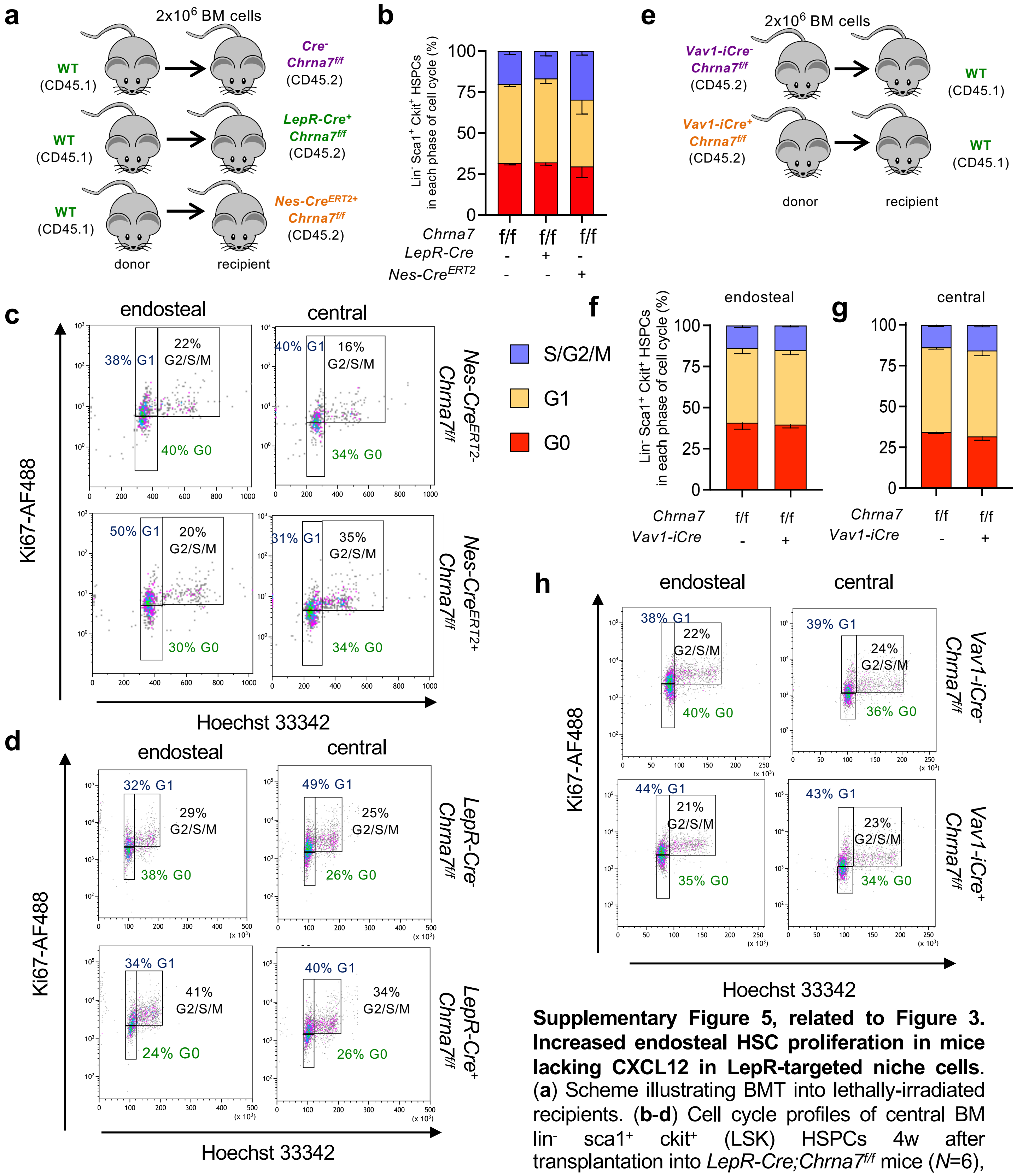

Supplementary Figure 5, related to Figure 3. Increased endosteal HSC proliferation in mice lacking CXCL12 in LepR-targeted niche cells. (**a**) Scheme illustrating BMT into lethally-irradiated recipients. (**b-d**) Cell cycle profiles of central BM lin⁻ sca1⁺ ckit⁺ (LSK) HSPCs 4w after transplantation into *LepR-Cre;Chrna7$^{f/f}$* mice (*N*=6), *Nes-Cre^{ERT2};Chrna7$^{f/f}$* mice (*N*=3) or control littermates (*N*=10 pooled from two independent experiments). **(c,d)** Representative flow cytometry diagrams showing ki67/Hoechst 33342 staining of endosteal or central BM HSPCs. The frequencies of the gated populations are indicated (**e**) Scheme illustrating BMT into lethally-irradiated recipients. (**f**,**g**) Cell cycle profiles of (**f**) endosteal and (**g**) central BM lin⁻ sca1⁺ ckit⁺ (LSK) HSPCs 4w after transplantation of *Vav1-iCre;Chrna7$^{f/f}$* mice or control littermates into lethally-irradiated WT CD45.1 mice (*N*=3). (**h**) Representative flow cytometry diagrams showing ki67/Hoechst 33342 staining of endosteal or central BM HSPCs. The frequencies of the gated populations are indicated. (**b**,**f**,**g**) Data are mean of biological replicates ± SEM. ANOVA and Tukey's multiple comparisons test.

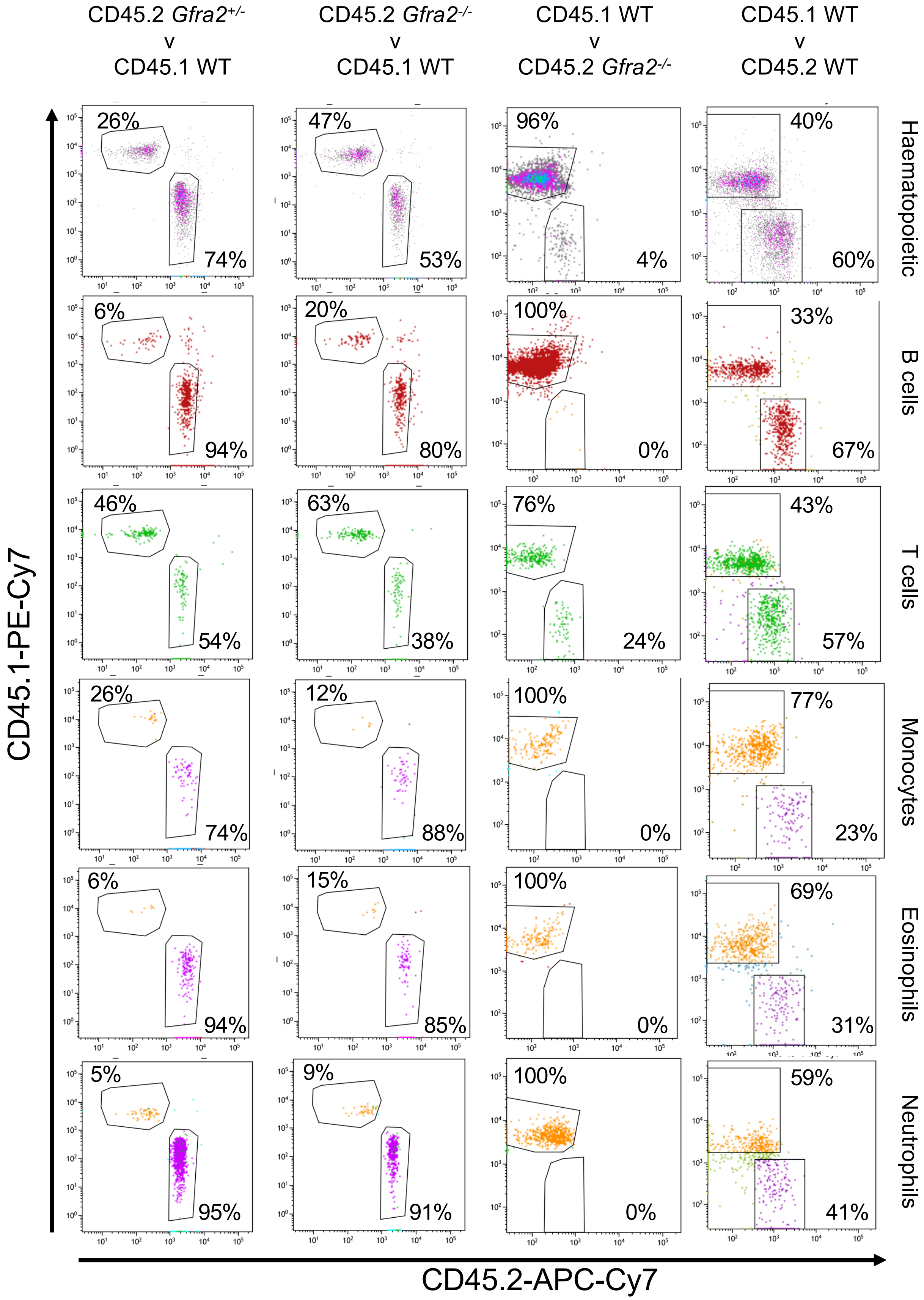

Supplementary Figure 6, related to Figure 4. Cholinergic-deficient niche compromises HSC quiescence, leading to increased reconstitution of primary recipients. Long-term transplantation of 500 endosteal lin$^-$sca1$^+$ckit$^+$ (LSK) CD150$^+$CD48$^-$ HSCs along with 10^5 BM helper cells into lethally-irradiated mice of indicated genotype (see scheme in Fig. 4a). Representative flow cytometry diagrams of multilineage haematopoietic chimerism 4 months after primary transplantation (*Gfra2*$^{+/-}$>CD45.1, *N*=5; *Gfra2*$^{-/-}$>CD45.1, *N*=6; CD45.1>*Gfra2*$^{-/-}$, *N*=4; CD45.1> *Gfra2*$^{+/+}$, *N*=4).

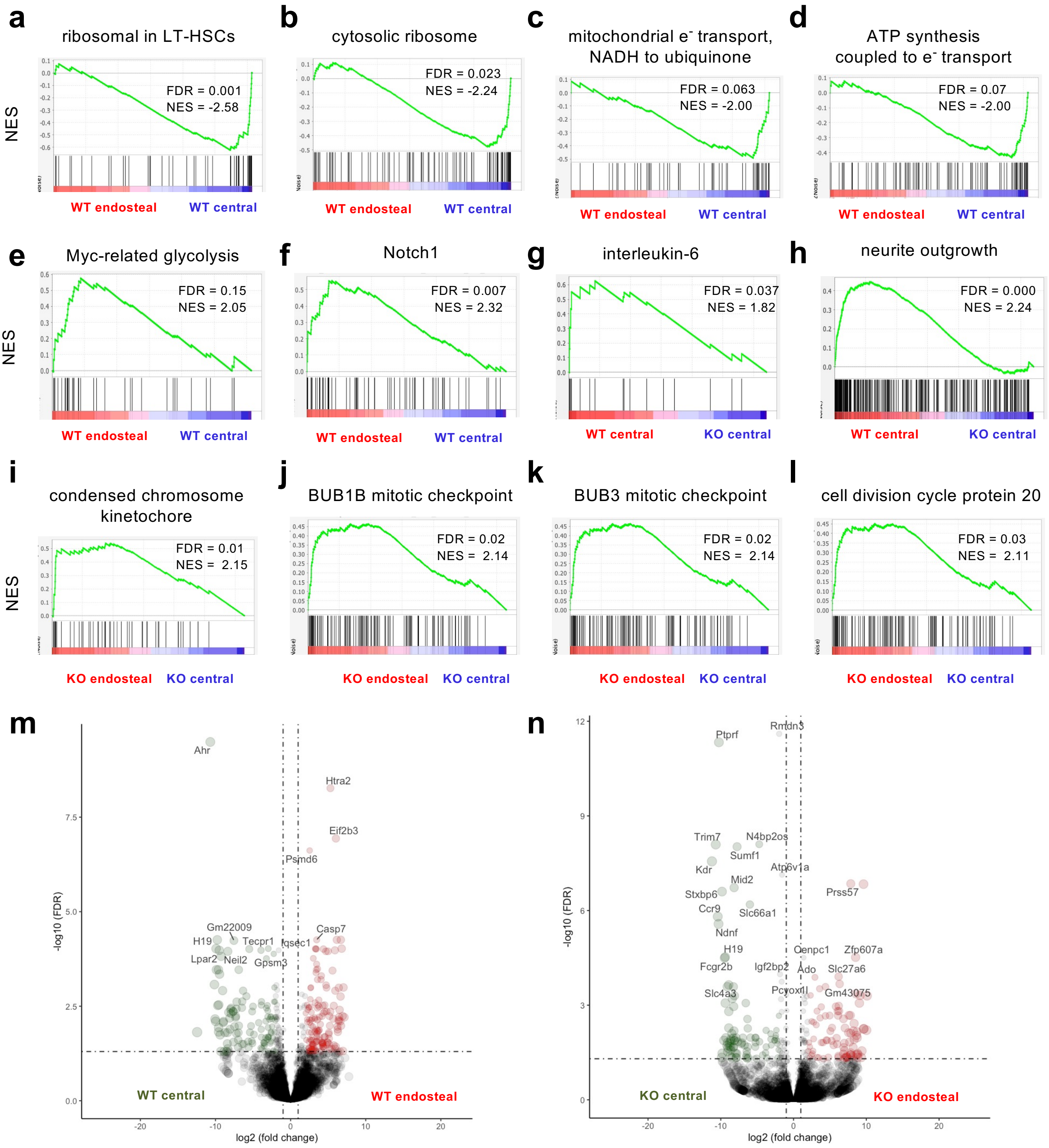

Supplementary Figure 7, related to Figure 5. Increased proliferation and deregulated neural-related pathways in HSCs from cholinergic-neural-deficient mice. Geneset enrichment analysis (GSEA) comparing endosteal and central BM HSCs of WT mice and *Gfra2*$^{-/-}$ mice (*N*=4 biological replicates). (**a-f**) Central (compared with endosteal) BM WT HSCs show increased ribosomal[74] (**a**,**b**) and mitochondrial respiration-related (**c**,**d**) pathways (GO), but reduced myc-related glycolysis and Notch1-associated pathways (**e**,**f**). (**g**,**h**) Reduced and IL6-dependent transcription[75] (**g**) and neurite outgrowth-related pathways (Biocarta) (**h**) in HSCs from *Gfra2*$^{-/-}$ mice. (**i-l**) Abnormal upregulation of gene sets associated with cell cycle progression and mitosis (GO and Reactome) in HSCs from endosteal *Gfra2*$^{-/-}$ BM. NES, normalized enrichment score; FDR, false discovery rate. (**m**,**n**) Volcano plots of differentially-expressed genes in HSCs from the central or endosteal BM of (**m**) WT and (**n**) *Gfra2*$^{-/-}$ mice.

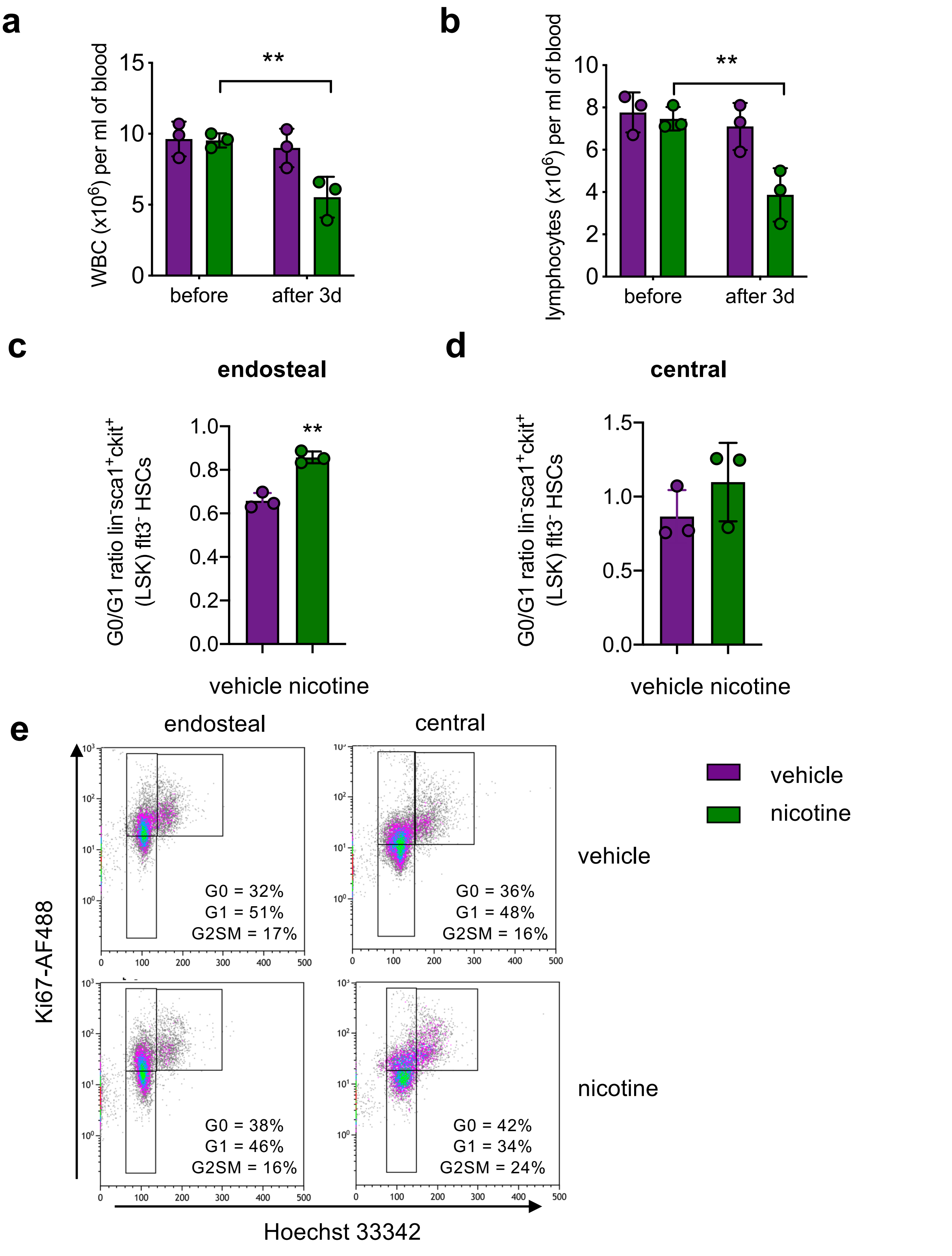

Supplementary Figure 8, related to Figure 7. Nicotine induces endosteal HSC quiescence. (**a**,**b**) White blood cells (WBC) (**a**) and circulating lymphocytes (**b**) in WT mice before (day 0) and 3 days after feeding mice with 1.5% saccharin in drinking water with/without 100µg/ml nicotine (*N*=3). ANOVA and Sidak's comparisons. (**c**,**d**) Ratio of quiescent HSCs (G0:G1 phases of the cell cycle) in the (**c**) endosteal or (**d**) central BM of WT mice treated with nicotine (100µg per ml of drinking water) over 3 days (*N*=3). Unpaired two-tailed *t* test. Each dot is a mouse. (**a-d**) Data are mean of biological replicates ± SEM. (**e**) Representative flow cytometry diagrams showing ki67/Hoechst 33342 staining in endosteal or central BM HSCs from WT mice treated with nicotine or vehicle. The frequencies of the gated populations are indicated.

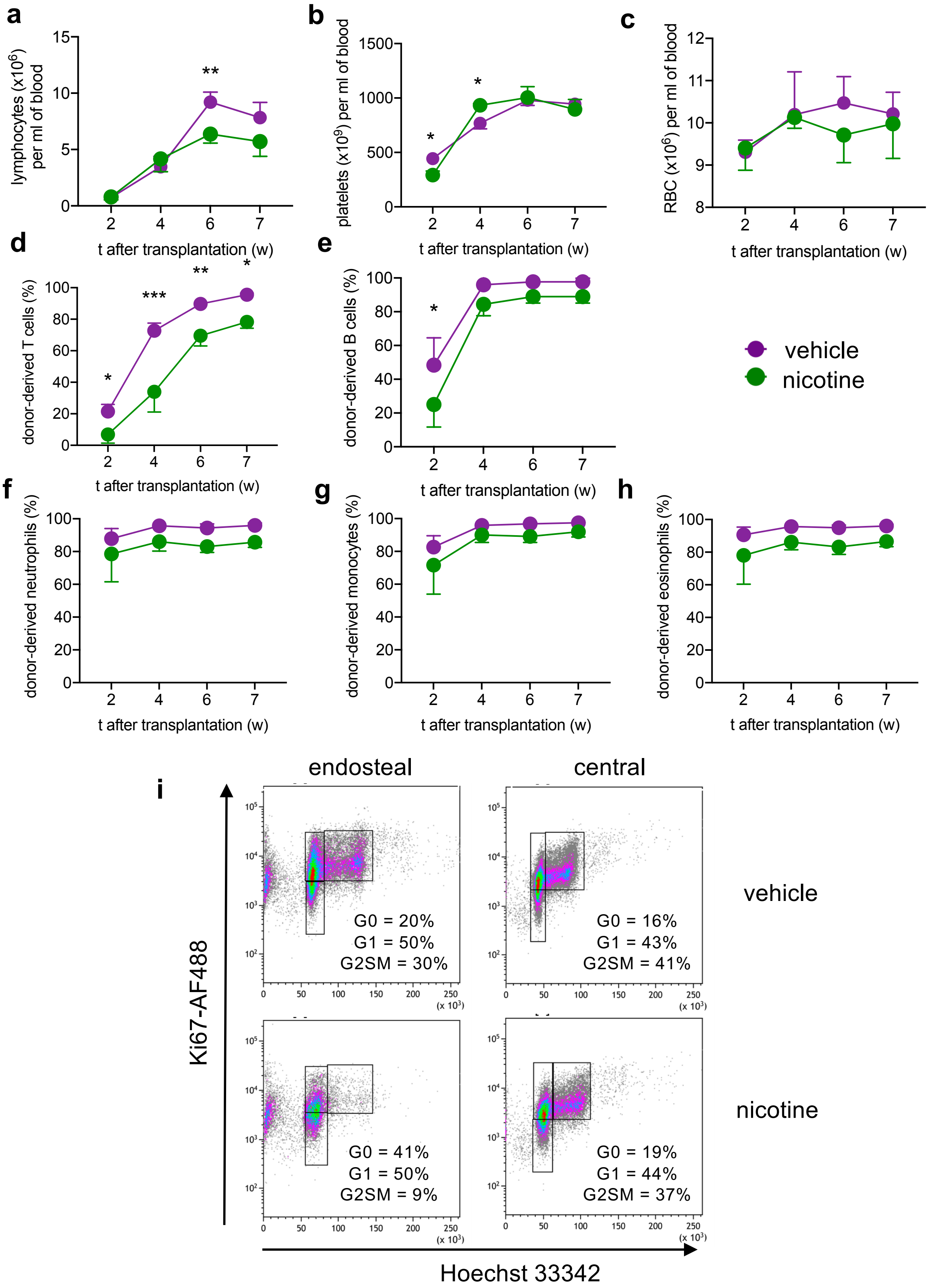

Supplementary Figure 9, related to Figure 7. Nicotine treatment induces HSC quiescence under stress. Sub-lethally irradiated WT CD45.2 recipients were transplanted with $2x10^6$ BM nucleated cells from CD45.1 mice and treated with 65µg/ml nicotine in their drinking water over 7 weeks. (**a-h**) Circulating lymphocytes (**a**), platelet (PLT) (**b**) and red blood count (**c**). Donor derived chimerism of T cells (**d**), B cells (**e**), neutrophils (**f**) monocytes (**g**), eosinophils (**h**) at specified time following transplantation (*N*=3). Data are mean of biological replicates ± SEM. *P < 0.05; **P < 0.01; ***P < 0.001. ANOVA and Sidak's comparisons. (**i**) Representative flow cytometry plots showing cell cycle distribution, determined by ki67/Hoechst 33342 staining, of lin^- $sca1^+$ $ckit^+$ $CD48^-$ $CD34^-$ $Flt3^-$ HSCs isolated from endosteal (bone) and central (marrow) BM fractions of nicotine treated or control mice 7w following transplantation. The frequencies of the gated populations are indicated.
