# Supplementary material for: Cholinergic signals preserve haematopoietic stem cell quiescence during regenerative haematopoiesis
Source: Nat Commun. 2022 Jan 27;13:543. doi: 10.1038/s41467-022-28175-1 (PMC8795384; doi:10.1038/s41467-022-28175-1)
Supplement: Supplementary file 3 — Description of Additional Supplementary Files [file 41467_2022_28175_MOESM3_ESM.pdf]

Description of Additional Supplementary Files

File Name: Supplementary Data 1
Description: **Comparison of HSC transcriptome between endosteal and central BM in WT mice.** Sample information, matrix of raw gene counts, normalized gene counts, differential gene expression analysis (Gene_DE_baseline) and gene set enrichment analysis (GSEA) showing pathways enriched in central (negative NES) or endosteal (positive NES) HSCs. NES, normalized enrichment score. FDR, False Discovery Rate. Kolmogorov Smirnov test.

File Name: Supplementary Data 2
Description: **Comparison of HSC transcriptome between endosteal and central BM in Gfra2$^{-/-}$ mice.** Sample information, matrix of raw gene counts, normalized gene counts, differential gene expression analysis (Gene_DE_baseline) and gene set enrichment analysis (GSEA) showing pathways enriched in central (negative NES) or endosteal (positive NES) HSCs. NES, normalized enrichment score. FDR, False Discovery Rate. Kolmogorov Smirnov test.

File Name: Supplementary Data 3
Description: **Patient characteristics.** AML, acute myeloid leukemia; MDS, myelodysplastic syndrome; ALL, acute lymphoblastic leukemia; MM, multiple myeloma; NHL, NonHodgkin lymphoma; HL, Hodgkin lymphoma; CLL, chronic lymphocytic leukemia; CMML, chronic myelomonocytic leukemia.

File Name: Supplementary Data 4
Description: The sequence of oligonucleotides used for quantitative real-time RT-PCR.
