# Supplementary material for: Cholinergic signals preserve haematopoietic stem cell quiescence during regenerative haematopoiesis
Source: Nat Commun. 2022 Jan 27;13:543. doi: 10.1038/s41467-022-28175-1 (PMC8795384; doi:10.1038/s41467-022-28175-1)
Supplement: Supplementary file 7 — Supplementary Data 4 [file 41467_2022_28175_MOESM7_ESM.pdf]

Supplementary Data 4. The sequence of oligonucleotides used for quantitative real-time RT-PCR.

**Gene**	**Forward**	**Reverse**
Cxcl12	CGCCAAGGTCGTCGCCG	TTGGCTCTGGCGATGTGGC
Nrtn	CAGCGGAGGCGCGTGCGCAGAGA	TAGCGGCTGTGCACGTCCAGGAA
ChAT	GCCAGTGGAAGAATCGTCAT	TTGTGCATGTGAGTGTGTGG
Gfra2	TTTAACATGATCTTGGCAAACG	AGCGGAGGGTTTCGTCTAA
Chrna3	ACTCCAAAAGCTGCAAGGAA	CAGAGCAGACAGGGACAACA
Chrna4	GCAGGGTCTCACAGGAAGAG	ACAGGATTTGGCTCCATCAC
Chrna5	CGCTCTTCTTCCACACACAA	TCTCGTGATGTAGCGAATGC
Chrna7	TTGTGCTGCGATATCACCAC	TTCATGCGCAGAAACCATGC
Chrnb2	GGGAAGATTATCGCCTCACA	GTGCAGTTCTGCTGGTCAAA
Chrnb4	GCTGAAGCTACGTCCACCTC	GAAGCTGACGCCCTCTAATG
Chrm1	CAGAAGTGGTGATCAAGATGCCTAT	GAGCTTTTGGGAGGCTGCTT
Chrm2	TGGAGCACAACAAGATCCAGAAT	CCCCTGAACGCAGTTTTCA
Chrm3	CCGCTCTACCTCTGTCCTTCA	GGTGATCTGACTTCTGGTCTTGAG
Chrm4	GTGACTGCCATCGAGATCGTAC	CAAACTTTCGGGCCACATTG
Chrm5	GGCCCAGAGAGAACGGAAC	TTCCCGTTGTTGAGGTGCTT
Gapdh	TGTGTCCGTCGTGGATCTGA	CCTGCTTCACCACCTTCTTGA
