# Supplementary material for: Cholinergic signals preserve haematopoietic stem cell quiescence during regenerative haematopoiesis
Source: Nat Commun. 2022 Jan 27;13:543. doi: 10.1038/s41467-022-28175-1 (PMC8795384; doi:10.1038/s41467-022-28175-1)
Supplement: Supplementary file 8 — Reporting Summary [file 41467_2022_28175_MOESM8_ESM.pdf]

nature research

Corresponding author(s): Simón Méndez-Ferrer

Last updated by author(s): Dec 10, 2021

Reporting Summary

Nature Research wishes to improve the reproducibility of the work that we publish. This form provides structure for consistency and transparency in reporting. For further information on Nature Research policies, see our Editorial Policies and the Editorial Policy Checklist.

Statistics

For all statistical analyses, confirm that the following items are present in the figure legend, table legend, main text, or Methods section.

n/a	Confirmed	
☐	☒	The exact sample size (*n*) for each experimental group/condition, given as a discrete number and unit of measurement
☐	☒	A statement on whether measurements were taken from distinct samples or whether the same sample was measured repeatedly
☐	☒	The statistical test(s) used AND whether they are one- or two-sided<br>*Only common tests should be described solely by name; describe more complex techniques in the Methods section.*
☐	☒	A description of all covariates tested
☐	☒	A description of any assumptions or corrections, such as tests of normality and adjustment for multiple comparisons
☐	☒	A full description of the statistical parameters including central tendency (e.g. means) or other basic estimates (e.g. regression coefficient) AND variation (e.g. standard deviation) or associated estimates of uncertainty (e.g. confidence intervals)
☒	☐	For null hypothesis testing, the test statistic (e.g. *F*, *t*, *r*) with confidence intervals, effect sizes, degrees of freedom and *P* value noted<br>*Give P values as exact values whenever suitable.*
☒	☐	For Bayesian analysis, information on the choice of priors and Markov chain Monte Carlo settings
☒	☐	For hierarchical and complex designs, identification of the appropriate level for tests and full reporting of outcomes
☒	☐	Estimates of effect sizes (e.g. Cohen's *d*, Pearson's *r*), indicating how they were calculated

Our web collection on statistics for biologists contains articles on many of the points above.

Software and code

Policy information about availability of computer code

Data collection	Pooled libraries of 4 replicates per sample were prepared using the Illumina Nextera XT DNA preparation kit. Amplified libraries were purified using Agencourt AMPure XP beads, quality-checked using Agilent high-sensitivity DNA chip on Agilent Bioanalyser 2100 and quantified using KAPA qPCR quantification kit (KAPA Biosystems, KK4824). Sequencing of 3 replicates per condition were performed on the Illumina Hi-Seq 4000 (single end, 50bp read length) by the CRUK Cambridge Institute Genomics Core facility
Data analysis	RNA-seq reads were aligned to Mus musculus genome (Ensembl version 38.81) using GSNAP (version 2015-09-29) with parameters (-B 5 –t 24 –n 1 –Q –N 1). Reads in features were counted with htweq-count (HTSeq version 0.5.3p3) with the parameter (-s no). Quality control was performed with the following cut-offs: more than one and a half million uniquely mappable reads, less than 20% of reads mapping to mitochondrial genes over mitochondrial + nuclear genes and more than 8500 high coverage genes identified. Counts were normalized using size factors as calculated by DESeq2 using a 10% FDR, and then log10 transformed. Highly variable genes were selected using the method described by Brennecke et al. PCA was then performed in R using the prcomp function.

For manuscripts utilizing custom algorithms or software that are central to the research but not yet described in published literature, software must be made available to editors and reviewers. We strongly encourage code deposition in a community repository (e.g. GitHub). See the Nature Research guidelines for submitting code & software for further information.

Data

Policy information about availability of data

All manuscripts must include a data availability statement. This statement should provide the following information, where applicable:

- Accession codes, unique identifiers, or web links for publicly available datasets
- A list of figures that have associated raw data
- A description of any restrictions on data availability

The data that support the findings of this study are included in the paper

Field-specific reporting

Please select the one below that is the best fit for your research. If you are not sure, read the appropriate sections before making your selection.

[x] Life sciences [ ] Behavioural & social sciences [ ] Ecological, evolutionary & environmental sciences

For a reference copy of the document with all sections, see nature.com/documents/nr-reporting-summary-flat.pdf

Life sciences study design

All studies must disclose on these points even when the disclosure is negative.

Sample size	The size of the mice cohorts were defined based on previous experience that the selected number of mice/samples should be sufficient to see an effect, taking into consideration the variability in the measurement. The sample size used in each experiment was not predetermined or formally justified for statistical power. Sample size for in vitro experiments were predetermined with sufficient replicates to validate experimental outcomes.
Data exclusions	N/A
Replication	All attempts to replicate the experiments presented were successful. Some experiments presented in this paper were reproduced by different researchers at least twice for mice studies and sometimes only one experiment was shown to facilitate the presentation of the data. Experiments that couldn't be reproduced were not included from the paper.
Randomization	The allocation of mice to be injected with a specific type of cells in each experiment has been done randomly. Other experimental groups were also randomly allocated
Blinding	The experiments were performed blindly

Reporting for specific materials, systems and methods

We require information from authors about some types of materials, experimental systems and methods used in many studies. Here, indicate whether each material, system or method listed is relevant to your study. If you are not sure if a list item applies to your research, read the appropriate section before selecting a response.

Materials & experimental systems

n/a	Involved in the study	
[ ]	[x]	Antibodies
[ ]	[x]	Eukaryotic cell lines
[x]	[ ]	Palaeontology and archaeology
[ ]	[x]	Animals and other organisms
[ ]	[x]	Human research participants
[x]	[ ]	Clinical data
[x]	[ ]	Dual use research of concern

Methods

n/a	Involved in the study	
[x]	[ ]	ChIP-seq
[ ]	[x]	Flow cytometry
[x]	[ ]	MRI-based neuroimaging

Antibodies

Antibodies used	The following antibodies were used: Rat Anti-Mouse CD45R/B220 (Clone RA3-6B2, BD Biosciences), Ckit (Clone 2B8, BioLegend), Biotin Mouse Lineage Panel (CD11b, Gr-1, Ter119, B220, CD3e) (BD Biosciences, 559971), Human/Mouse GFR alpha -2/GDNF R alpha -2 Antibody (Bio-Techne, AF429), CD45.1 (Clone A20, Insight Biotechnology), CD45.2 (Clone 104, BioLegend UK Ltd), Sca 1 (Clone SQ19, BD Biosciences), Ckit (Clone 2B8, BioLegend UK Ltd), Goat anti rabbit AF488 (Thermo Fisher Scientific, A-11008), Sca 1 PE Cy7 (Clone E13-161.7, BioLegend UK Ltd), CD34 (Clone HM34, BioLegend UK Ltd), CD150 (Clone TC15-12F12.2, BioLegend UK Ltd), CD48 (Clone HM48-1, BioLegend UK Ltd), CD41 (Clone MWReg30, BD Pharmingen), CD49b (Clone HMa2, BD Biosciences), CD48 (Clone HM48-1, BioLegend UK Ltd), CD135 (Clone A2F10, BioLegend UK Ltd), Flt3 (Clone A2F10.1, BD Biosciences), Rabbit anti ki67 (Abcam, AB15580), CD31 (Clone MEC13.3, BD Biosciences), Hoechst 33342 Solution (20 mM) (Thermo Fisher Scientific, 62249), Dylight 650

Donkey anti rat (Thermo fisher scientific, SA5-10029), Ly-6G/Ly-6C (Gr1) (Clone HK1.4, Biolegend), CD11b (Clone M1/70, Biolegend), CD3e (Clone 145-2C11, Insight Biotechnology). Biotinylated antibodies were detected with fluorochrome-conjugated streptavidin (BD Biosciences).All antibodies were used at 1:200 except for biotinylated lineage antibody mix at 1:100, cells incubated in 300ul for staining.

Validation

Antibodies were all purchased and validated by the supplier

Eukaryotic cell lines

Policy information about cell lines

Cell line source(s)	MS-5 cells (DSMZ, ACC 441), MC3T3-E1 cells (ATCC CRL-2593), ST-2 and MLO-Y4 cells were kindly provided by Prof. Lynda Bonewald
Authentication	*Describe the authentication procedures for each cell line used OR declare that none of the cell lines used were authenticated.*
Mycoplasma contamination	Cell lines were regularly tested for mycoplasma
Commonly misidentified lines (See ICLAC register)	NA

Animals and other organisms

Policy information about studies involving animals; ARRIVE guidelines recommended for reporting animal research

Laboratory animals	Age and sex-matched Gfra2-/-REF12, Nes-gfp61 (generously provided by G.E. Enikolopov), FVB/N-Adrb3tm1Lowl/J (Stock number 006402), B6;129X1-Nrtntm1Jmi/J (Stock number 012238), B6.129S7-Chrna7tm1Bay/J (Stock number 003232), ChATBAC-eGFP (Stock number 007902), α7nAChRflox (Stock number 026965), B6.129(Cg)-Leprtm2(cre)Rck/J (Stock number 008320), Nes-creERT2(REF 62) (generously provided by G. Fishell), B6.Cg-Commd10Tg(Vav1-icre)A2Kio/J (Stock number 008610) (Jackson Laboratories), and congenic CD45.1 and CD45.2 C57BL/6J mice (Charles River) were used in this study. For genetic lineage tracing, B6.Cg-Gt(ROSA)26Sortm14(CAG-tdTomato)Hze/J (Ai14D) reporter mice (Stock number 007908) were crossed with B6.129S-Chattm1(cre)Lowl/MwarJ mice (Chat-IRES-Cre) (Stock number 031661) and Wnt1-Cre2 (Stock number 022501) (Jackson Laboratories). Mice were housed in specific pathogen free facilities. All experiments using mice followed protocols approved by the Animal Welfare Ethical Committees at the University of Cambridge (PPL 70/8406 and PPL P0242B783). All experiments were compliant with EU recommendations.
Wild animals	This study didn't involve any wild animal.
Field-collected samples	This study didn't involve samples collected from the field.
Ethics oversight	All experiments using mice followed protocols approved by the Animal Welfare Ethical Committees at the University of Cambridge (PPL 70/8406 and PPL P0242B783). All experiments were compliant with EU recommendations.

Note that full information on the approval of the study protocol must also be provided in the manuscript.

Human research participants

Policy information about studies involving human research participants

Population characteristics	N/A
Recruitment	N/A
Ethics oversight	N/A

Note that full information on the approval of the study protocol must also be provided in the manuscript.

Flow Cytometry

Plots

Confirm that:

- [x] The axis labels state the marker and fluorochrome used (e.g. CD4-FITC).
- [x] The axis scales are clearly visible. Include numbers along axes only for bottom left plot of group (a 'group' is an analysis of identical markers).
- [x] All plots are contour plots with outliers or pseudocolor plots.
- [x] A numerical value for number of cells or percentage (with statistics) is provided.

Methodology

Sample preparation	All samples' preparation required for this study are specified in the Methods.

Instrument	All instruments used in this study are specified in the Methods : we used the LSRFortessa flow cytometer (BD Biosciences, Franklin Lakes, NJ) and the sorter (FACS Aria cell sorter, BD Bioscience) equiped with FACSDiva Software (BD Biosciences).
Software	Data were analysed using Kaluza software (Beckman Coulter).
Cell population abundance	PDGFRα+Sca1- skeletal stem cells (SSC)- 0.03%<br>PDGFRα+Sca1+ (PαS) cells- 0.03%<br>PDGFRα-CD51+Sca1+ bone-lining osteoprogenitors (OPCs)- 0.04%<br>PDGFRα-CD51+Sca1- osteoblast precursors (OBPs)- 0.07%
Gating strategy	The gating strategy for all the relevant populations described in this study is described in the Methods.

☒ Tick this box to confirm that a figure exemplifying the gating strategy is provided in the Supplementary Information.
